# Multimodal consumer choice prediction using EEG signals and eye tracking

**DOI:** 10.3389/fncom.2024.1516440

**Published:** 2025-01-08

**Authors:** Syed Muhammad Usman, Shehzad Khalid, Aimen Tanveer, Ali Shariq Imran, Muhammad Zubair

**Affiliations:** ^1^Department of Computer Science, Bahria School of Engineering and Applied Science, Bahria University, Islamabad, Pakistan; ^2^Department of Computer Engineering, Bahria School of Engineering and Applied Science, Bahria University, Islamabad, Pakistan; ^3^Department of Creative Technologies, Air University, Islamabad, Pakistan; ^4^Department of Computer Science, Norwegian University of Science and Technology, Gjøvik, Norway; ^5^Interdisciplinary Research Center for Finance and Digital Economy, King Fahd University of Petroleum and Minerals, Dhahran, Saudi Arabia

**Keywords:** EEG, eye tracking, neuromarketing, CNN-LSTM, multimodal

## Abstract

Marketing plays a vital role in the success of a business, driving customer engagement, brand recognition, and revenue growth. Neuromarketing adds depth to this by employing insights into consumer behavior through brain activity and emotional responses to create more effective marketing strategies. Electroencephalogram (EEG) has typically been utilized by researchers for neuromarketing, whereas Eye Tracking (ET) has remained unexplored. To address this gap, we propose a novel multimodal approach to predict consumer choices by integrating EEG and ET data. Noise from EEG signals is mitigated using a bandpass filter, Artifact Subspace Reconstruction (ASR), and Fast Orthogonal Regression for Classification and Estimation (FORCE). Class imbalance is handled by employing the Synthetic Minority Over-sampling Technique (SMOTE). Handcrafted features, including statistical and wavelet features, and automated features from Convolutional Neural Network and Long Short-Term Memory (CNN-LSTM), have been extracted and concatenated to generate a feature space representation. For ET data, preprocessing involved interpolation, gaze plots, and SMOTE, followed by feature extraction using LeNet-5 and handcrafted features like fixations and saccades. Multimodal feature space representation was generated by performing feature-level fusion for EEG and ET, which was later fed into a meta-learner-based ensemble classifier with three base classifiers, including Random Forest, Extended Gradient Boosting, and Gradient Boosting, and Random Forest as the meta-classifier, to perform classification between buy vs. not buy. The performance of the proposed approach is evaluated using a variety of performance metrics, including accuracy, precision, recall, and F1 score. Our model demonstrated superior performance compared to competitors by achieving 84.01% accuracy in predicting consumer choices and 83% precision in identifying positive consumer preferences.

## 1 Introduction

Neuromarketing, a dynamic fusion of neuroscience and marketing, has emerged through the innovative use of non-invasive Brain–Computer Interface (BCI) technology, revolutionizing the concept of marketing. Marketing is a connection between production and consumers. A good product can fail to target its desired audience without effective marketing (Assel, 1995). To create products and services with the highest profit potential, it is crucial to thoroughly understand consumer behavior and develop a corresponding advertising strategy. This requires a comprehensive understanding of the buyer's decision-making process, which typically includes need recognition, information search, evaluation, purchase decision, and post-purchase behavior (Armstrong et al., 2014; Peter et al., 1999; Vecchiato et al., 2011). Researchers have employed Electroencephalography (EEG) and Eye Tracking (ET) to analyze the brain activity and gaze outcomes when exposed to different stimuli for several decades.

EEG is a technique used to assess the electrical activity within a person's cranial structure. This involves placing numerous electrodes on the scalp, a method known as scalp EEG. It is particularly preferred for recording brain waves because it is simple and does not involve any invasive procedure, while other methods are preferable because they are efficient in monitoring brain activity (Teplan, 2002; Fisch, 1999). It records changes in electrical activity and oscillations within the brain. The amplitude of the signals are proportional to the type of mental activity experienced when exposed to stimuli (Homan et al., 1987). Eye tracking, on the other hand, involves gathering information on visual attention through the capturing of eye movements. The eye tracking revealed where and for how long a person looked at the different elements, whereas EEG can uncover the emotional and cognitive response elicited by these stimuli.

Neuromarketing, a multidisciplinary field at the intersection of neuroscience, psychology, and economics, explores the complex dynamics of how advertisements can significantly impact product sales. Unlike traditional marketing research methods such as interviews, reviews, and questionnaires, neuromarketing seeks to surpass the limitations inherent in these approaches. These conventional methods often fall short of fully revealing consumers' insights toward products, as individuals may encounter challenges in conveying their preferences or may be hesitant to express them comprehensively. Moreover, the chances of data manipulation add a layer of complexity to the reliability of findings.

Human behavior is influenced by processes operating beneath the conscious threshold. In response to these challenges, neuromarketing offers a revolutionary shift, going beyond direct questions about products and exploring the deeper subconscious areas of consumers' minds. The essence is to get insights in a non-invasive manner, extracting authentic preferences and choices that may outstand conventional probing techniques. It offers a deeper and more precise insight into consumer behavior. This leads to the development of innovative and successful marketing tactics, ultimately driving increased sales. In the expansive and intricate landscape of the advertising industry, where expenditures vary based on geographical location, industry sector, and individual company strategies. The main contributions of this research study are as follows:

- A novel multimodal framework has been proposed, integrating EEG signals and eye-tracking data to enhance consumer preference prediction. This approach combines the strengths of both modalities, addressing the lack of sufficient multimodal research in the domain.
- A robust feature extraction pipeline has been designed, combining handcrafted features and automated features derived through deep learning. This hybrid approach provides a more comprehensive representation of the data, bridging an identified gap in the existing literature.
- Ensemble classification techniques have been proposed to address the challenges of class imbalance and improve prediction accuracy. By utilizing multiple classifiers and optimizing their integration, significant improvements in performance metrics were achieved compared to traditional methods.

## 2 Literature review

Many individuals are often reserved in expressing their complete thoughts and preferences during product evaluation, creating a challenge in comprehending the complexities of consumer decision-making. The emergence of neuroimaging tools provides a quick and convenient method to understand a customer's brain activity when evaluating and choosing different products. Consumer choice recognition typically involves three pivotal stages. The initial step encompasses preprocessing, wherein unwanted noise is eliminated from both EEG and ET signals. Following this, relevant features are extracted, and subsequently, EEG and ET signals are classified based on consumer preferences. In neuromarketing studies, the recording of both EEG and ET data equips researchers to get into the complex interplay of factors that influence how the human psyche makes choices among different products.

### 2.1 Predictive approaches for consumer preference based on EEG signals

Researchers have proposed multiple methods for classification between like vs. dislike for neuromarketing in recent years. A typical method consists of preprocessing the EEG signals and extracting the features followed by the classification. Researchers have used various preprocessing techniques employed in predicting consumer preferences. Bandpass filtering, widely utilized for EEG signal noise reduction in numerous studies (Murugappan et al., 2014; Alimardani and Kaba, 2021; Aldayel et al., 2021; Georgiadis et al., 2022, 2023a), serves as a prominent technique. Independent Component Analysis (ICA) has been adopted by researchers to eliminate noise in their proposed methods (Aldayel et al., 2021; Georgiadis et al., 2022; Telpaz et al., 2015; Hakim et al., 2021). Telpaz et al. (2015) and Hakim et al. (2021) have also applied the Notch Filter for preprocessing. Downsampling, an effective method employed by several researchers like (Aldayel et al., 2021), proves valuable for reducing the sampling rate of EEG data. Moreover, the Savitzky–Golay filter was utilized to effectively remove artifacts (Aldayel et al., 2021; Yadava et al., 2017; Shah et al., 2022). Murugappan et al. (2014) applied the Surface Laplacian Filter, and Kumar et al. (2019) used high and low pass filters for the purpose of preprocessing EEG signals.

After the preprocessing of EEG signals, the extraction of features is pivotal for classifying likes and dislikes. Many approaches are employed for feature extraction like LSTM (Shah et al., 2022). Telpaz et al. (2015) have leveraged N200, or N2, is an event-related potential (ERP) component. The Power Spectrum Density (PSD) provides the distribution of power across diverse frequencies in the signal (Murugappan et al., 2014; Alimardani and Kaba, 2021; Shah et al., 2022). Similarly, Discrete Wavelet Transform (DWT) (Arif et al., 2023) introduces a process of iteratively breaking down the signal into approximation and detail coefficients across multiple scales, a technique adeptly utilized by researchers for feature extraction (Aldayel et al., 2021; Yadava et al., 2017; Shah et al., 2022; Kumar et al., 2019). Aldayel et al. (2021) have contributed by employing Welch Method. This metric, corresponding to the spatial standard deviation, offers insights into the amount of activity at each time point in the potential field. EEG signals are represented as Sample Covariance Matrices (SCMs) that are measured entities scattered over a particular Riemannian manifold by Georgiadis et al. (2022, 2023a). One of the most commonly used method is to analyze EEG data is to break the signal into functionally distinct frequency bands. Telpaz et al. (2015) and Hakim et al. (2021) extracted frequency bands to extract features from EEG signals. These features provide high interclass variance which is useful in accurate classification. The details of these various features are briefly described in the following table understanding what kind of preprocessing techniques and feature extraction methods were used in this research, as shown in Table 1.

**Table 1:** Comparison of existing consumer preference prediction methods using EEG signals.

**References**	**Data**	**Year**	**Preprocessing**	**Feature extraction**	**Classifier**	**Accuracy (%)**
Murugappan et al. (2014)	EEG	2014	Bandpass filter Surface Laplacian filter	PSD SE SC	kNN PNN	96.62
Telpaz et al. (2015)	EEG	2015	Notch filter
ICA	ERSP N200 (ERP)	Random	59 65
Yadava et al. (2017)	EEG	2017	Savitzky-Golay	DWT	HMM	70.33
Aldayel et al. (2021)	EEG	2021	Downsampling Bandpass filter ICA Savitzky-Golay	DWT Welch method	DNN SVM kNN RF	83 81 73 87
Alimardani and Kaba (2021)	EEG	2021	Bandpass filter	PSD	CNN EC (SVM RF, LOG)	74.57 63.5
Hakim et al. (2021)	EEG	2021	Notch filter ICA	FBP Hemispheric symmetry	SVM LOG kNN DT	68.51
Shah et al. (2022)	EEG	2022	Savitzky-Golay FFT SMOTE	DWT PSD LSTM	EC (SVM, DT, DNN)	96.89
Georgiadis et al. (2022)	EEG	2022	Bandpass filter ICA	SCM	SVM Ensemble	73.11
Georgiadis et al. (2023a)	EEG	2023	Bandpass Filter	SCM	SPDNet	72.18

There are simple features such as the frequency distribution of words to parametric and non-parametric features, etc. for classification between the “like” and “dislike” classes. Statistical features in the time domain include the mean average, variance/ standard deviation, skewness, and kurtosis. Also, frequency domain features such as moments of spectrum like spectral centroid, variational coefficients, and even skewness in the spectrum can be incorporated. In addition, other techniques of dimensionality reduction such as Principal Component Analysis (PCA) have also been used in this study by the researchers to extract features and to reduce dimensionality. Table 1 can give a brief idea of these various features and present an outline of the most important preprocessing proposals and the feature extraction applied in the present research.

Deep Neural Network (DNN), Support Vector Machine (SVM), Random Forest (RF), and *k*-Nearest Neighbors (kNN) resulted a maximum accuracy of 87%. Alimardani and Kaba (2021) proposed an ensemble classifier based on SVM, RF, Logistic Regression(LOG) and Convolution Neural Network(CNN). Murugappan et al. (2014) applied for *k*NN and Probabilistic Neural Network (PNN) for classification of EEG signals. Hakim et al. (2021) conducted a comprehensive study utilizing EEG, focusing solely on Machine Learning algorithms and acheived an accuracy of 68.51%. Shah et al. (2022) predicted users' preferences for advertisements using an ensemble classifier [SVM, Decision Tree (DT), DNN] achieving an impressive accuracy of 96.89%. Yadava et al. (2017) presented the first dataset of neuromarketing. This dataset featured stimuli in the form of images of commercial products, labeled as either “like” or “dislike,” and Hidden Markov Model (HMM) was employed for the classification of EEG signals based on likes and dislikes. Georgiadis et al. (2022) applied a SVM Ensemble including three SVM classifiers, while their research (Georgiadis et al., 2023a) used architecture of SPDNet. It is a deep learning architecture designed for processing data that lie on Symmetric Positive Definite (SPD) matrices.

### 2.2 Predictive approaches for consumer preference based on EEG signals and ET data

Researchers have employed various techniques to preprocess EEG signals and ET data for understanding consumer preferences. Khushaba et al. (2013) utilized a combination of ICA and DWT for EEG signal preprocessing. Matukin et al. (2016) and Samsuri et al. (2016) incorporated band-pass filtering in their methodologies. Christoforou et al. (2017) downsampled EEG data and applied a Notch filter to mitigate DC drifts. For processing pupil dilation signals, Slanzi et al. (2017) employed linear interpolation followed by band-pass filtering. Mashrur et al. (2024) adopted the Automatic Subspace reconstruction functionality from EEGLAB for noise reduction, subsequently applying a notch filter at 50 Hz to suppress power line artifacts.

Matukin et al. (2016) applied Fast Fourier Transform (FFT) to derive features from EEG signals. Samsuri et al. (2016) utilized P300 and N100 components for EEG signal analysis, while employing Pupil Dilation features for eye-tracking data. Christoforou et al. (2017) introduced the Attentional-asynchrony metric based on the Eye-Gaze Divergence Index and used epoched EEG measurements to formulate a Cognitive-congruency aggregate metric. Slanzi et al. (2017) employed Principal Component Analysis (PCA) to extract features from EEG signals. Garćıa-Madariaga et al. (2019) focused on Alpha Band Oscillations for EEG signals and Area of Interest (AOI) for eye-tracking data. Mashrur et al. (2024) categorized features into three domains: time domain (TD), frequency domain (FD), and time-frequency domain (TFD), subsequently employing a classifier for optimal feature selection.

Table 2 provides comparative analysis of existing methods of neuromarketing based on EEG and ET. Khushaba et al. (2013) used mutual information analysis that indicated important factors affecting the buying decision. Samsuri et al. (2016) measured the attention levels of users when observing an advertisement through the use of EEG and ET signals. In the study by Christoforou et al. (2017), the R2 metric was employed to assess the predictive capability of the suggested neural and eye-tracking metrics on the box office success of films. Slanzi et al. (2017), aimed to determine the sections of a webpage that were most probable to attract clicks through the application of Logistic Regression. Mashrur et al. (2024) used the SVM classifier is used with RBF kernel for classifying strong and weak preference EEG signals attaining an accuracy of 97%.

**Table 2:** Comparison of existing consumer preference prediction methods using EEG and ET data.

**Author**	**Data**	**Year**	**Preprocessing**	**Feature extraction**	**Classification**	**Results**
Khushaba et al. (2013)	EEG ET	2013	ICA DWT	FFT	Mutual Information	Preference
Matukin et al. (2016)	EEG ET	2016	Bandpass filter	FFT	Not specified	Improvement in Ads
Samsuri et al. (2016)	EEG ET	2016	Bandpass filter	P300 ERP N100 Pupil dilation	Statistics	ERP and the ET results were inconsistent
Christoforou et al. (2017)	EEG ET	2017	Downsampling Notch filter	Attent.Asynchrony Cogn. Congruency	Regression R2	72% accuracy
Slanzi et al. (2017)	EEG ET	2017	Interpolation BandPass filter	PCA	Logistic regression	71.09% accuracy
Garćıa-Madariaga et al. (2019)	EEG ET	2019	Not specified	Alpha-Band Oscillation AOI	Not specified	Eye movements could predict packaging preference.
Mashrur et al. (2024)	EEG ET	2023	ASR Notch filter	TD FD TFD	SVM-RBF	96.97% accuracy

Following research gaps have been identified after a comprehensive literature review of both EEG and ET consumer preference prediction methods:

- There is a lack of sufficient multimodal research investigating the combined effectiveness of EEG and eye-tracking.
- The issue of class imbalance remains a significant challenge in this field.
- The integration of handcrafted and automated features in a combined feature set has not been much explored.
- The limited use of ensemble learning methods represents a notable research gap.

## 3 Dataset

The NeuMa dataset (Georgiadis et al., 2023b) has been used for this particular research work and this comprises of 42 participants who were all Greek speakers; 23 males and 19 females. The dataset is made up of 144 supermarket products and this is presented in six brochure pages whereby each brochure is made up of 24 products. Targets were highlighted by users with a left-click of the mouse on products of interest. Consequently, each of the subjects has two files for every subject, which contains the records of their interactions with the products.

Table 3 provides brief description of the NeuMa dataset. Subjects were positioned at an arm's length or 50 cm away from the screen which is a 28 inch LCD monitor, and navigation on the digital brochure page and choice of products with the left click of the mouse. For the page navigation arrow keys of the keyboard were used. Every subject's data set involved EEG and eye-tracking information and mouse clicks and positions. There are EEG signals and eye movements, mouse clicks, and cursor movements collected in the given dataset. Among these data streams, currently only EEG and ET type data streams are being used.

**Table 3:** Summary of NeuMa dataset.

**Attribute**	**Details**
Number of subjects	42 (23 males, 19 females)
Number of products	144
Number of pages	6 (24 products per page)
Average selections	18 products per participant
Data files per subject	2 (S01.xdf, S01.xls)
EEG device	Wearable Sensing DSI24
EEG sampling frequency	300 Hz
EEG sensors	21 dry sensors
ET device	Tobii pro fusion
ET sampling frequency	120 Hz

**Figure 1:**
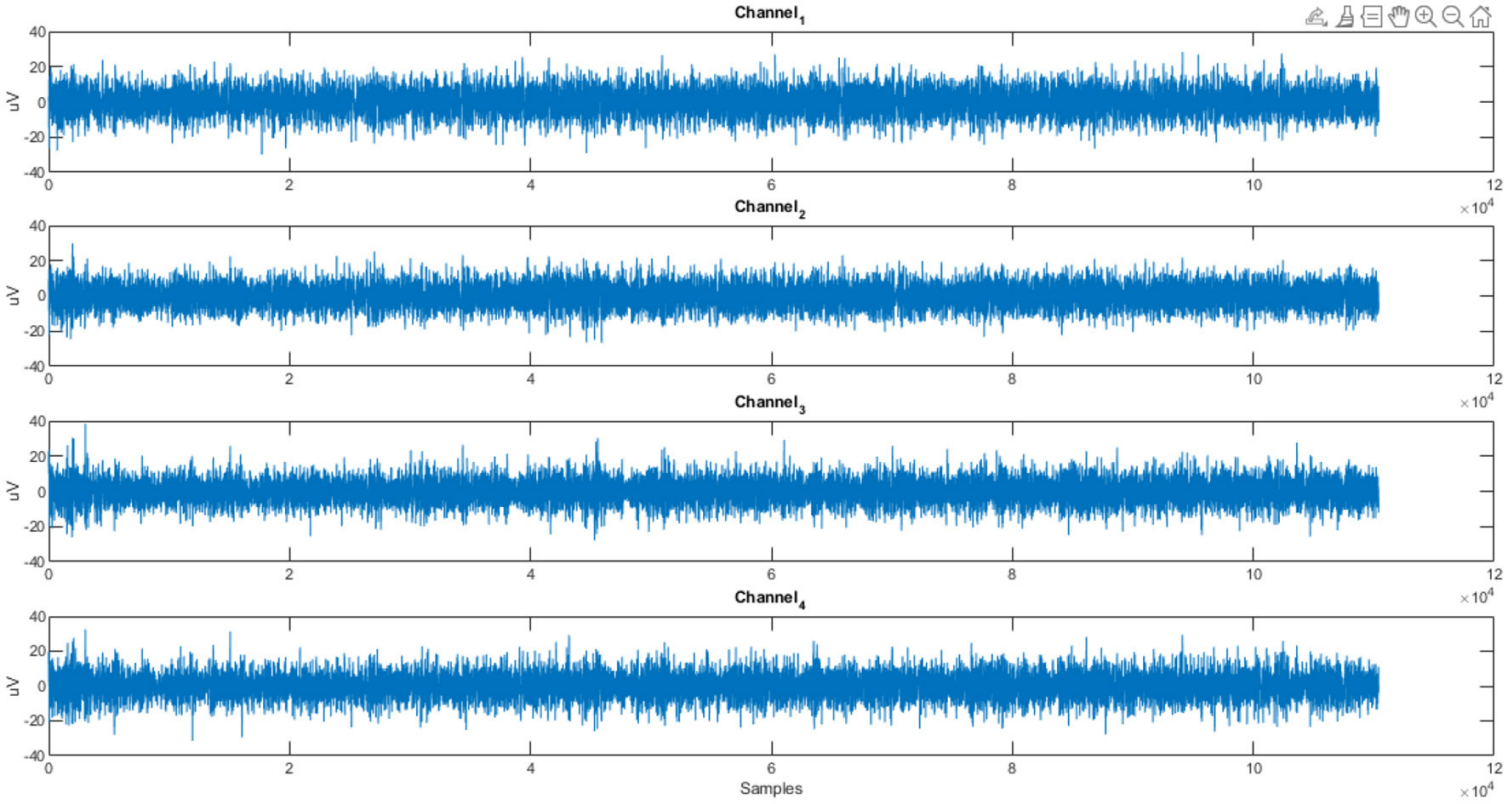
Customer response (Subject S01): EEG data stream for product (NeuMa dataset: EEG data capturing the cerebral activity of a subject for a product).

**Figure 2:**
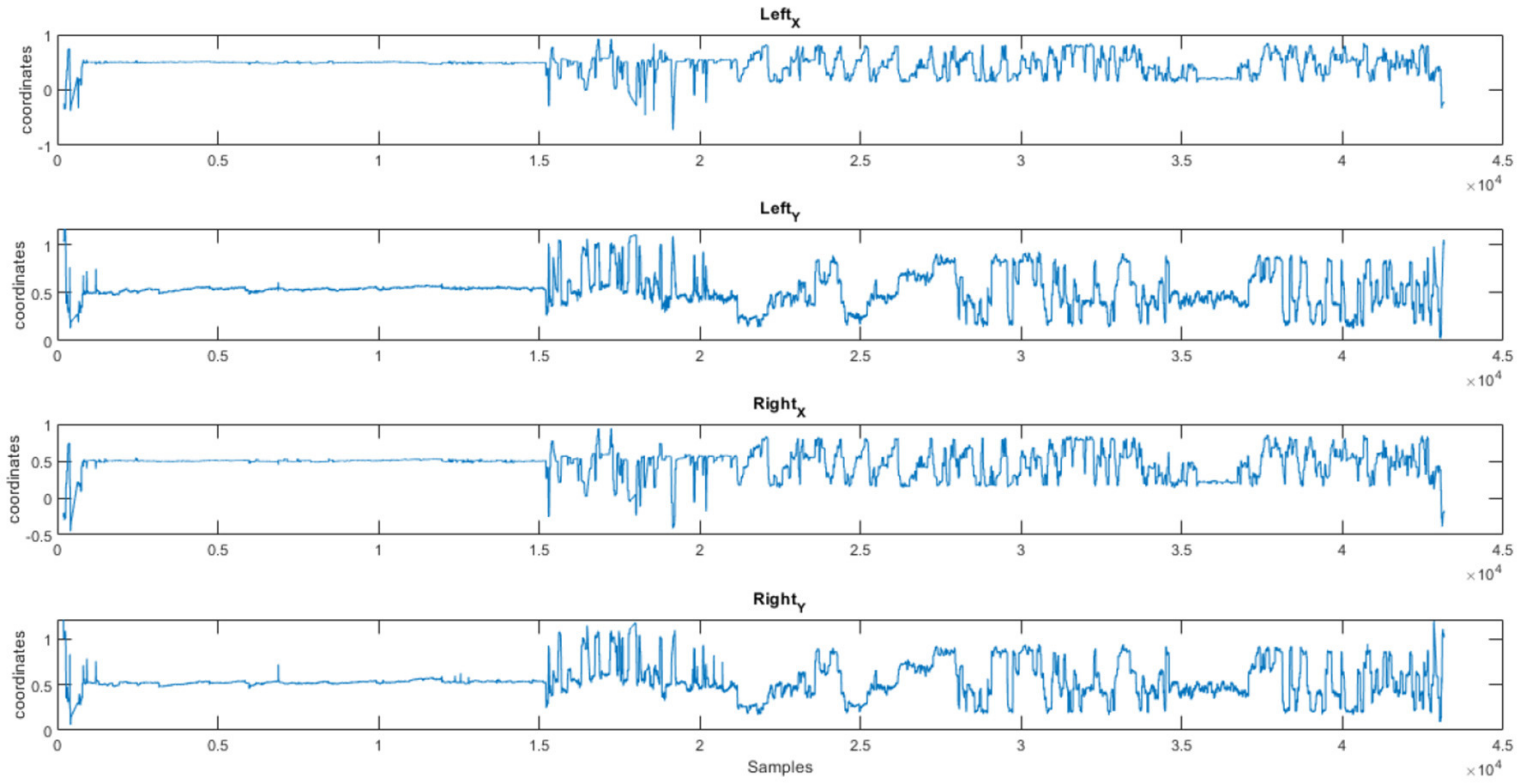
Customer response (Subject S01): eye tracking coordinates (*X, Y*) for a product (NeuMa dataset: ET data revealing the gaze pattern linked to a product).

**Figure 3:**
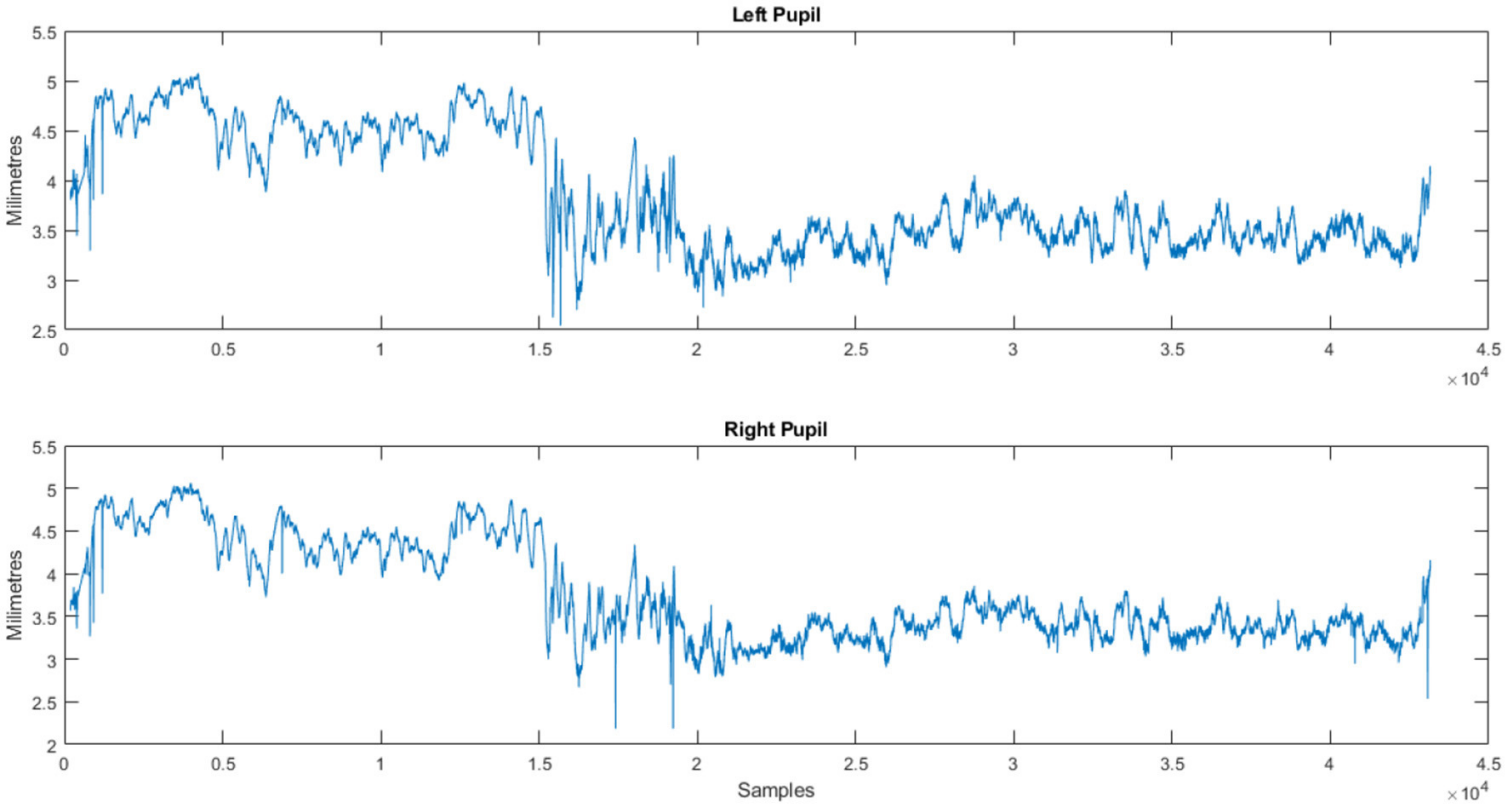
Customer response (Subject S01): pupil dilation stream for a product (NeuMa dataset: ET data revealing pupil dilation patterns linked to a product).

After the experiment, participants filled in a questionnaire containing demographic details about the individuals, profiling details about the participants as well as about the products provided to them like personality profile, tendency to indulge in impulse buying and about the products given to them like reasons for selection of product, familiarity with the product and frequency for buying the product. EEG data was recorded by DSI 24 system with the sampling rate of 300 Hz from 21 electrodes. This eye-tracking data was at a sampling rate of 120Hz and the Tobii Pro Fusion eye-tracker was used to collect the data. Figures 1–3 show the plots of EEG, ET, and Pupil dilation data, respectively (Tobii, 2024; Georgiadis et al., 2023b).

## 4 Methodology

**Figure 4:**
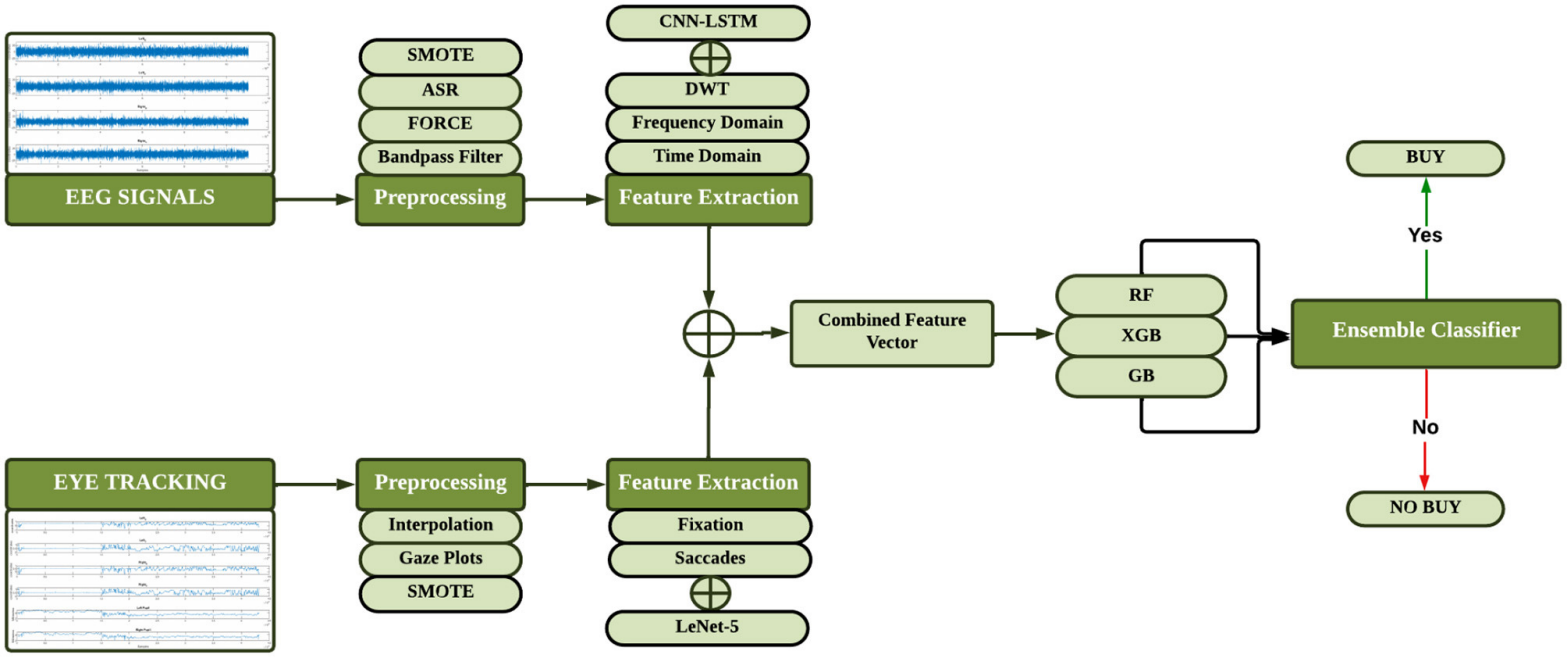
Flow diagram of proposed methodology.

Proposed method consists of three steps: EEG and ET signal preprocessing, feature extraction and classification. The pre-processing of the EEG signals is done with the help of Bandpass Butterworth filter (0.5–45Hz), Artifact Subspace Reconstruction (ASR) and the Fast Orthogonal Regression for Classification and Estimation (FORCE). Signals are then split in segments overlapping each other since the data amount is at a manageable size. In the same manner, preprocessing of Eye Tracking (ET) data; missing values are eliminated/taken care of using a linear interpolation and the data is segmented using overlapping window techniques. Non-technique based features are derived from the EEG and ET signals using statistical and frequency domain analysis The technique incorporated is CNN-LSTM for EEG and LeNet5 for ET data. First, for each input modality, feature-level fusion is used to combine these extracted features, and second, improvements are made to classification using both manually defined and learned features are used. Figure 4 displays the flow diagram of the proposed methodology.

### 4.1 Preprocessing of EEG signals

Electroencephalogram (EEG) signals are often contaminated with various types of noise, including muscle activity, eye movements, and electrical interference from other devices. To analyze EEG data effectively, preprocessing steps such as filtering are crucial. Electroencephalography (EEG) signals require preprocessing to remove noise and isolate frequencies of interest. One method is applying a bandpass filter with band range 0.5–45 Hz.

In order to filter out Signals with artifact in the EEG data a band pass filter was employed together with Artifact Subspace Reconstruction (ASR). ASR also helps in eradicating interferences like shrugs and blinks and leaves the signal's quality intact for analysis. This technique is very important in neuromarketing research as it offers clean signal filtration yet preserves the original signal. Fast method for Orthogonal Regression for Classification and Estimation EEG sounds are done using orthogonal basis vector and FORCE for the preprocessing of the data. It is applied to remove noise while improving the quality of the signal, making it suitable for the situations that require fast and accurate artifacts detection. Specifically, the signals from the EEG signals were band-pass filtered and then analyzed by ASR and FORCe to obtain the best results.

The overlapping window technique again divides the filtered signals to get more detailed data and make the signals continuous. The division of the continuous EEG signals make it more manageable and this was achieved by gaining small samples of 300 Hz with the window size being one second with 300 data entries. The overlapping of the windows has the advantage of achieving greater density of information and continuity of the signal.

### 4.2 Preprocessing of eye tracking data

Linear interpolation is a technique of curve fitting in which a straight line is drawn between two points to give the estimated point. It handles missing data if eye-tracking signals due to long blinking are missing using what is known as the straight-line interpolation method. Due to the ability to replace a missing value with approximated data samples that occur before and after the gap, a continuous signal is achieved. This step is necessary for preserving the quality of eye-tracking signal and further analysis of study subjects' attention and eye movement behavior.

As for removing missing values in the eye-tracking dataset, linear interpolation has been applied, the next step of the data preprocessing is the data segmentation based on the overlapping window. This ensures that the maximum amount of information is collected as the windows have a 50% overlap in which every two consecutive windows have 50% of the same data points. This rises the density of data and contributes to non-fragmentation of signal which helps in maintaining coherency. The splitting of records further improves difference detection or comparison which is made possible by the window size of one second and a sampling rate of 120 Hz; this means that each segment's data set has 120 data points.

**Figure 5:**
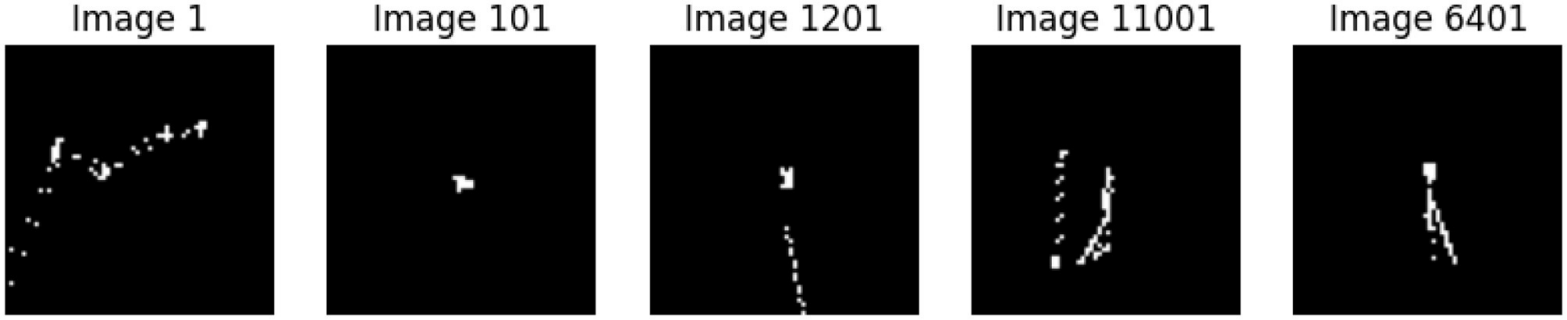
A few gaze plots of ET data.

Gaze plots are basically eye movement data obtained through eye tracking displayed graphically as data points. They are developed by placing fixation areas on a graph of the observed stimulus. For the movements of the eyes, the *X* and *Y* coordinates are transformed into the 64 × 64 canvas where the black background implies no gaze while the white point marks a gaze. These points are the coordinates of the location on the canvas, and the original gaze plot images are saved, converted to the NumPy array and then to grayscale for analysis. Figure 5 presents various Gaze plots.

**Figure 6:**
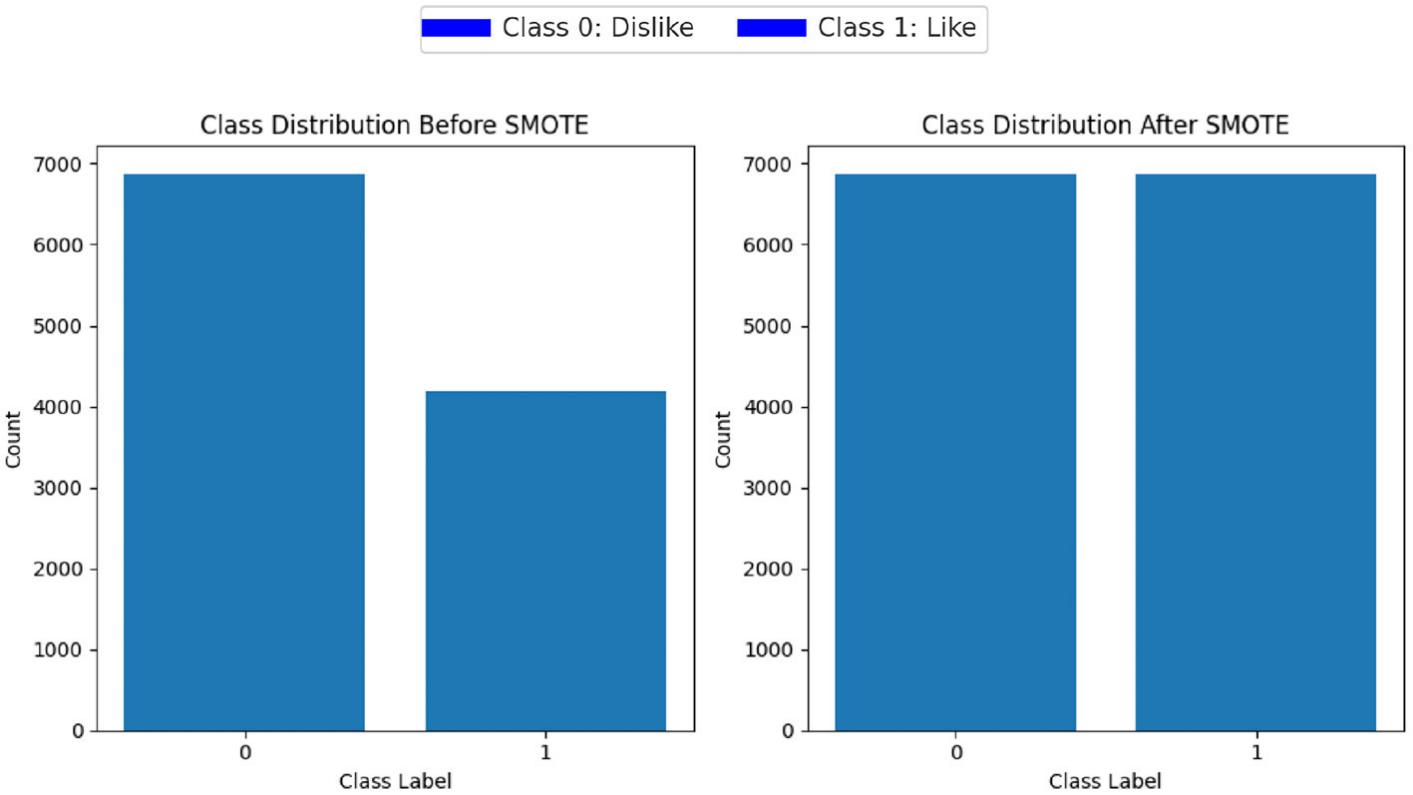
Class distribution before and after SMOTE.

Class imbalance refers to situations where one class (the minority class) is significantly underrepresented compared to another class (the majority class). This class imbalance can lead to biased models that perform poorly on the minority class. To address the issue of class imbalance within the dataset, we used Synthetic Minority Over-sampling Technique (SMOTE) (Chawla et al., 2002). SMOTE works by generating synthetic examples of the minority class to balance the class distribution. The process involves creating new instances of minority class samples by interpolating between existing minority class samples SMOTE first identifies the minority class samples in the dataset. For each minority class sample of EEG and ET data, SMOTE selects its k nearest neighbors in the feature space. The value of k we chose is 3, as it was giving the best results. For each minority class sample, SMOTE generates synthetic samples along the line segments connecting it to its k nearest neighbors. The number of synthetic samples created for each minority class sample is determined by a specified oversampling ratio. By creating synthetic samples, SMOTE increases the representation of the minority class in the dataset, balancing the class distribution. Figure 6 displays the class distribution before and after application of SMOTE.

### 4.3 Feature extraction of EEG signals

After preprocessing the EEG data, which typically involves filtering out noise and artifacts, the next step is to extract meaningful features from the cleaned data. Feature extraction transforms the raw EEG signals into a set of representative features that can be used for further analysis, such as classification. We used a few common statistical features include Mean, Variance, Skewness, and Kurtosis.

The mean of the EEG signal provides a measure of the central tendency of the signal. It indicates the average value of the signal over a specified period.


(1) $$\begin{array}{l} \mu =\frac{1}{N}\overset{N}{\underset{i=1}{\sum }}x_{i} \end{array}$$

Where *N* is the number of data points and *x*_*i*_ represents the EEG signal values.

The co-efficient of variation is used to determine the spread of the signal value in relation to the mean of the EEG signals. That reveal information about the fluctuation in the activity of the brain.


(2) $$\begin{array}{l} \sigma^{2}=\frac{1}{N}\overset{N}{\underset{i=1}{\sum }}{\left(x_{i}-\mu \right)}^{2} \end{array}$$

Skewness indicates the extent of probability distribution of the EEG signals asymmetrical nature. The absolute value of skewness is >1, <1 or zero if the distribution is highly skewed to the right, left, or symmetric respectively.


(3) $$\begin{array}{l} \gamma_{1}=\frac{N}{\left(N-1\right)\left(N-2\right)}\overset{N}{\underset{i=1}{\sum }}{\left(\frac{x_{i}-\mu }{\sigma }\right)}^{3} \end{array}$$

Kurtosis quantifies the degree of the two at both the center and the tails of the probability density function of the EEG signal. It also implies that the data contains some outliers.


(4) $$\begin{array}{l} \gamma_{2}=\frac{N\left(N+1\right)}{\left(N-1\right)\left(N-2\right)\left(N-3\right)}\overset{N}{\underset{i=1}{\sum }}{\left(\frac{x_{i}-\mu }{\sigma }\right)}^{4}-\frac{3{\left(N-1\right)}^{2}}{\left(N-2\right)\left(N-3\right)} \end{array}$$

Welch's Method (Welch, 1967) is one of the robust and standard method to estimate power spectral density (PSD) of a signal. Even if it splits the signal into overlapping sections, then they apply a function known as windowing on sections, calculate the periodogram of each segment, and finally the averages these periodograms. In this feature, extraction was performed for all the EEG channels, considering the average power of the given signal within all possible frequency bands. This feature quantifies the amplitude deviations of the power from the energy of the signal at various frequency bands. The wavelet transform is the process by which a signal is broken down in different parts that are localized both temporally and in the frequency domain. Since, mean of DWT coefficients gives the average value of the coefficients, we obtained the mean of this parameter. This feature calculates the extent of fluctuations valued in the domain of wavelet coefficients.

Finally, Statistical features and frequency domain features and wavelet transform features are then combined to construct an information vector for each sample that will serve as the input to the model. The technical advantage implemented in the feature set uses time-frequency characteristic as well as multi-resolution analysis.

After removing noise from the EEG signals, features were extracted for the “Buy” and “No Buy” classes using two common approaches: handcrafted feature extraction and automated feature extraction via deep learning techniques. In the handcrafted approach, features are extracted without considering the class of the EEG signals. In contrast, automated feature extraction leverages deep learning models like Convolutional Neural Networks (CNNs) (LeCun et al., 1998) and Long Short-Term Memory (LSTM) networks (Hochreiter and Schmidhuber, 1997), which consider the class of the EEG signals during feature extraction. This method can lead the class of the EEG signals during feature extraction. This method can lead to improved classification performance due to lower intraclass variance and higher interclass variance.

LSTMs are a type of recurrent neural network (RNN) that are capable of learning long-term dependencies (Shah et al., 2022). They have a chain-like structure with repeating modules. The core of the LSTM module consists of a cell state, and three gates to regulate the flow of information: the input gate, forget gate, and output gate. For feature extraction from EEG signals, we implement a convolutional neural network LSTM architecture. The CNN takes the segmented time-domain signals it has: the CNN's inputs are the number of EEG channels and temporal segments of signals. Convolutional layers perform spatial features extraction using filters of particular sizes which are succeeded by the max-pooling layers in an attempt to decrease the dimension and hence increasing the efficiency of the training process. The features from the CNN layers are flattened and reshaped so as to be fed into LSTM layer that takes into consideration temporality of the data. CNN and LSTM are combined because the former analyses the spatial information of the signals while the latter analyses the temporal information of the signals making it appropriate to classify the EEG signals. Table 4 provides a summary of our proposed CNN-LSTM model.

**Table 4:** Summary of proposed CNN-LSTM model.

**Layer**	**Output shape**	**Parameters**
Input layer	(None, 19, 300, 1)	0
Conv2D	(None, 17, 298, 32)	320
Max pooling	(None, 8, 149, 32)	0
Conv2D	(None, 6, 147, 64)	18,496
MaxPooling	(None, 3, 73, 64)	0
Flatten layer	(None, 14,016)	0
Reshape layer	(None, 14,016, 1)	0
LSTM	(None, 64)	16,832
Dense	(None, 128)	8,320
Dense	(None, 64)	8,256

### 4.4 Feature extraction of ET data

After preprocessing ET data and handling the class imbalance issue features are extracted from it. Similar to feature extraction from EEG data, statistical features can be employed to quantify various aspects of these movements.

#### 4.4.1 Fixation duration

Fixation duration represents the average time a user spends fixating on a specific Area of Interest (AOI) and is analogous to the mean in EEG analysis. It provides insight into the level of attention paid to that area.

#### 4.4.2 Saccade amplitude

Saccade amplitude is just like variance in EEG, its calculates the distance between one fixation to another fixation. Large value of saccade amplitude represents jump from one fixation to other.

We applied the LeNet-5 (LeCun et al., 1998) model, a foundational CNN architecture developed by Yann LeCun, originally designed for image recognition tasks like classifying handwritten digits. The model processes input images through convolutional layers with filters to extract features, followed by max-pooling layers to reduce dimensionality. After multiple convolution and pooling layers, the feature maps are flattened into a vector for the classification layers. This structure effectively captures spatial features in the data, making it suitable for image recognition tasks. Here's an explanation for the LeNet-5 model summarized in Table 5.

**Table 5:** Summary of LeNet-5 model.

**Layer**	**Output shape**	**Parameters**
Input layer	(None, 64, 64, 1)	0
Conv2D	(None, 60, 60, 6)	456
Max pooling	(None, 30, 30, 6)	0
Conv2D	(None, 26, 26, 16)	2,416
MaxPooling	(None, 13, 13, 16)	0
Flatten layer	(None, 2,704)	0

Features extracted from EEG signals and ET data are concatenated to form a combined feature vector with a size of 16,720, which is then fed into an ensemble classifier. Optimizer used is Adam and loss function used is Mean Squared Error. Adam is a gradient-based optimization algorithm. Its update rules are given by:

**Update Rules:**


(5) $$\begin{array}{l} m_{t}=\beta_{1}m_{t-1}+\left(1-\beta_{1}\right)g_{t}, \end{array}$$

(6) $$\begin{array}{l} v_{t}=\beta_{2}v_{t-1}+\left(1-\beta_{2}\right)g_{t}^{2}, \end{array}$$

(7) $$\begin{array}{l} {\hat{m}}_{t}=\frac{m_{t}}{1-\beta_{1}^{t}},\; \end{array}$$

(8) $$\begin{array}{l} {\hat{v}}_{t}=\frac{v_{t}}{1-\beta_{2}^{t}},\; \end{array}$$

(9) $$\begin{array}{l} \theta_{t}=\theta_{t-1}-\eta \frac{{\hat{m}}_{t}}{\sqrt{{\hat{v}}_{t}}+\epsilon },\; \end{array}$$

where:

- *m*_*t*_: First moment (mean of gradients),
- *v*_*t*_: Second moment (uncentered variance of gradients),
- *g*_*t*_: Gradient at time step *t*,
- β_1_, β_2_: Exponential decay rates for the moment estimates,
- η: Learning rate,
- ϵ: Small constant to prevent division by zero,
- θ_*t*_: Parameters at time step *t*.

The Mean Squared Error loss function is given by:


(10) $$\begin{array}{l} \text{MSE}=\frac{1}{n}\overset{n}{\underset{i=1}{\sum }}{\left(y_{i}-{ŷ}_{i}\right)}^{2}, \end{array}$$

where:

- *n*: Number of data points,
- *y*_*i*_: True value for the *i*-th data point,
- ŷ_*i*_: Predicted value for the *i*-th data point.

### 4.5 Ensemble classifier

After pre-processing and feature extraction, the final step is classification which is performed to categorize the sample as Buy vs. Non-buy. We have used three stacking ensemble classification approach in which features are first passed to three different classifiers including Random Forest, Gradient Boosting and XGBoost. Prediction obtained from these three classifiers is then stacked to get the final classification. Random Forest has beeen used as meta model in the stacking ensemble (Wolpert, 1992).

#### 4.5.1 Base classifiers

Random Forest (RF): Random Forest grows a whole forest during training. It is a bagging technique in which every tree makes a prediction and then make a final prediction. This classifier is good to handle high dimensional data which is often the case with EEG and ET features (Breiman, 2001)


(11) $$\begin{array}{l} T_{k}\left(x\right)=\text{Class label predicted by the }k\text{-th tree.} \end{array}$$

(12) $$\begin{array}{l} P_{\text{RF}}\left(x\right)=\text{Majority Vote}\left\{T_{1}\left(x\right),T_{2}\left(x\right),\ldots ,T_{K}\left(x\right)\right\} \end{array}$$

Gradient Boosting (GB): This is a strong method that constructs decision trees iteratively where each stage used in identifying the mistakes committed by the prior trees. Friedman (2001). The final prediction is:


(13) $$\begin{array}{l} P_{\text{GB}}\left(x\right)=\overset{M}{\underset{m=1}{\sum }}\alpha_{m}h_{m}\left(x\right) \end{array}$$

where:

- *h*_*m*_(*x*): the *m*-th weak learner,
- α_*m*_: the weight of the *m*-th learner.

XGBoost (XGB): XGBoost is an optimized version of Gradient Boosting (Chen and Guestrin, 2016). The prediction for XGBoost is:


(14) $$\begin{array}{l} P_{\text{XGB}}\left(x\right)=\overset{M}{\underset{m=1}{\sum }}\eta_{m}h_{m}\left(x\right)+\text{Ω}\left(h_{m}\right) \end{array}$$

where:

- η_*m*_: learning rate,
- Ω(*h*_*m*_): regularization term.

### 4.6 Meta-classifier

The meta-classifier takes the outputs of the base classifiers as input. In this case, a Random Forest is used as the meta-classifier.

In the first step, predictions are collected from the base classifiers for the training dataset:


(15) $$\begin{array}{l} P_{\text{RF}}\left(x\right),\;P_{\text{GB}}\left(x\right),\;P_{\text{XGB}}\left(x\right). \end{array}$$

(16) $$\begin{array}{l} Z=\left[\begin{array}{ccc} P_{\text{RF}}\left(x_{1}\right) & P_{\text{GB}}\left(x_{1}\right) & P_{\text{XGB}}\left(x_{1}\right)\\ P_{\text{RF}}\left(x_{2}\right) & P_{\text{GB}}\left(x_{2}\right) & P_{\text{XGB}}\left(x_{2}\right)\\ \vdots & \vdots & \vdots \\ P_{\text{RF}}\left(x_{n}\right) & P_{\text{GB}}\left(x_{n}\right) & P_{\text{XGB}}\left(x_{n}\right) \end{array}\right] \end{array}$$

In the final step, meta classifiers is trained to get the final classification result on *Z*. We have used Random Forest as meta classifier.


(17) $$\begin{array}{l} P_{\text{Meta}}\left(x\right)=\text{Meta-RF}\left(Z\right). \end{array}$$

For unseen data *x*, the stacking ensemble works as follows: Each base model makes a prediction:


(18) $$\begin{array}{l} P_{\text{RF}}\left(x\right),\;P_{\text{GB}}\left(x\right),\;P_{\text{XGB}}\left(x\right). \end{array}$$

These predictions form a new feature vector for *x*:


(19) $$\begin{array}{l} Z_{x}=\left[\begin{array}{c} P_{\text{RF}}\left(x\right),P_{\text{GB}}\left(x\right),P_{\text{XGB}}\left(x\right) \end{array}\right]. \end{array}$$

The meta-classifier uses *Z*_*x*_ to make the final prediction:


(20) $$\begin{array}{l} P_{\text{Final}}\left(x\right)=P_{\text{Meta}}\left(x\right). \end{array}$$

Base model outputs are as follows:


$$\begin{array}{l} \;P_{\text{RF}}(x)=\text{Random Forest prediction},\\ \;P_{\text{GB}}(x)=\text{Gradient Boosting prediction},\\ P_{\text{XGB}}(x)=\text{XGBoost prediction}. \end{array}$$

Meta-model (Random Forest) output is as follows:


(21) $$\begin{array}{l} P_{\text{Meta}}\left(x\right)=\text{Majority Vote}\left\{T_{k}\left(Z_{x}\right)\right\},\;k=1,2,\ldots ,K. \end{array}$$

Final stacking ensemble prediction:


(22) $$\begin{array}{l} P_{\text{Final}}\left(x\right)=P_{\text{Meta}}\left(x\right). \end{array}$$

### 4.7 Hyperparameters optimization

For the machine learning models, Random Forest Classifier was set with 265 estimators for the Optuna (Akiba et al., 2019) tuned model and 100 for the Stacking Classifier final estimator. The Gradient Boosting Classifier uses 89 estimators and the XGB Classifier is set with 300 estimators. These three estimators are surrounded by the Stacking Classifier such that the Random Forest classifier is used as the final estimator. Further, the imbalance of the data is tackled using SMOTE with the specified random state of 42. To split the dataset into cross-validation, the keyword Stratified K-Fold is used with the parameter setting of the number of folds as 10, shuffle as True, and random state as 42.

In the case of the deep learning models used in automatic feature extraction, the CNN connected with the LSTM is applied for the feature extraction of the EEG data. The structure of the model consists of an LSTM layer with 64 neurons and dense layers with 128 and 64 neurons, optimizer used is Adam and loss function used is Mean Squared Error. For the eye-tracking data the LeNet-5 is employed; it consists of two dense layers with 120 and 84 units and a sigmoid layer is used at the output for binary classification. This model is trained with the Adam optimizer with binary cross-entropy as the loss function and accuracy as the parameter over 50 epochs. The data splitting involves a train test split of 80–20 and further division of the remaining data in equal proportions to validate and test the model.

#### 4.7.1 Stratified cross-validation

Once we were set up, with the ensemble classification pipeline formulated, the next logical step was to assess its utility. To this end, the strategy used was a rigorous method known as the stratified 10-fold cross-validation (Kohavi, 1995). However, stratified cross-validation goes one step further than this as it guarantees the resultant folds as having the same proportion of classes as those of the original data-set.

## 5 Results and discussion

The efficiency of classification models is evaluated in terms of the metrics that measure the ability of the ML algorithm to classify the objects appropriately. Selecting the appropriate metrics is essential for achieving an accurate and objective assessment and measuring performance in such problems with skewed classes or different costs associated with an error. Accuracy for the most basic performance indicator that show the number of instances out of all the data that belong to the correct class. Precision also known as positive predictive value, measures the proportion of true positives among all predicted positives. It reflects how often the model correctly identifies a positive case.

Specificity test evaluates the proportion of actual negatives which are correctly identified by the model as negative, while, recall or sensitivity evaluates the proportion of actual positives which are correctly identified by the model as positive. They indicate how well the model captures all the positive instances in relation to the available training examples. F1 score is an average of recall and precision that yields proportional insights into both these measures. It's particularly useful when both false positives and false negatives are equally undesirable. It's particularly useful when both false positives and false negatives are equally undesirable.

Table 6 represents the quantitative comparison of the employed methods, namely accuracy, precision rate, recall, and F1 score. The proposed method achieves the highest accuracy of 0.84, significantly outperforming the other methods. The improvement in accuracy can be attributed to the effective integration of ML and DL features along with the stacking ensemble technique. The precision of the proposed method (0.83) indicates its superior ability to correctly identify positive instances compared to other methods. This is particularly important in reducing false positives, which is critical in applications where the cost of false positives is high. For recall the proposed method gives 0. 84 which shows that the proposed method is also good in the recall sense it captures most of the true positive instances. Large recall component means that the model is going to include many more positives into the result set at the cost of possibly including negative instances, which is particularly important where false negatives are undesirable. The proposed method claims to achieve an F1 score of 0.83 which balances the precision and recall rates of identifying fishes with equal importance. This is because F1 score is a harmonic mean of precision and recall and a high F1 score indicates that the propose method is both precise and accurate, studied and tested on different measure standards and tables.

**Table 6:** Evaluation metrics for different methods.

**Method**	**Accuracy**	**Precision**	**Recall**	**F1 score**
1- EEG (not preprocessed, ML features, SVM)	0.62	0.60	0.59	0.59
2- EEG (preprocessed, DL features, SVM + RF)	0.65	0.61	0.64	0.62
3- EEG (preprocessed, ML + DL features, SVM + RF + DT)	0.74	0.70	0.75	0.72
4- ET (not preprocessed, ML features, RF)	0.60	0.58	0.59	0.59
5- ET (preprocessed, DL features, DT)	0.62	0.55	0.56	0.55
6- ET (preprocessed, ML + DL features, XGB + RF + DT)	0.65	0.64	0.61	0.62
7- EEG & ET (not preprocessed, ML features, SVM)	0.72	0.71	0.67	0.68
8- EEG & ET (preprocessed, DL features, RF)	0.75	0.74	0.72	0.73
9- EEG & ET (preprocessed, ML + DL features, SVM + RF + XGB)	0.80	0.78	0.79	0.78
**Proposed- EEG & ET (preprocessed, ML + DL features, stacking ensemble)**	**0.84**	**0.83**	**0.84**	**0.83**

Ablation study has been performed and bold values show the results obtained from final methodology.

**Figure 7:**
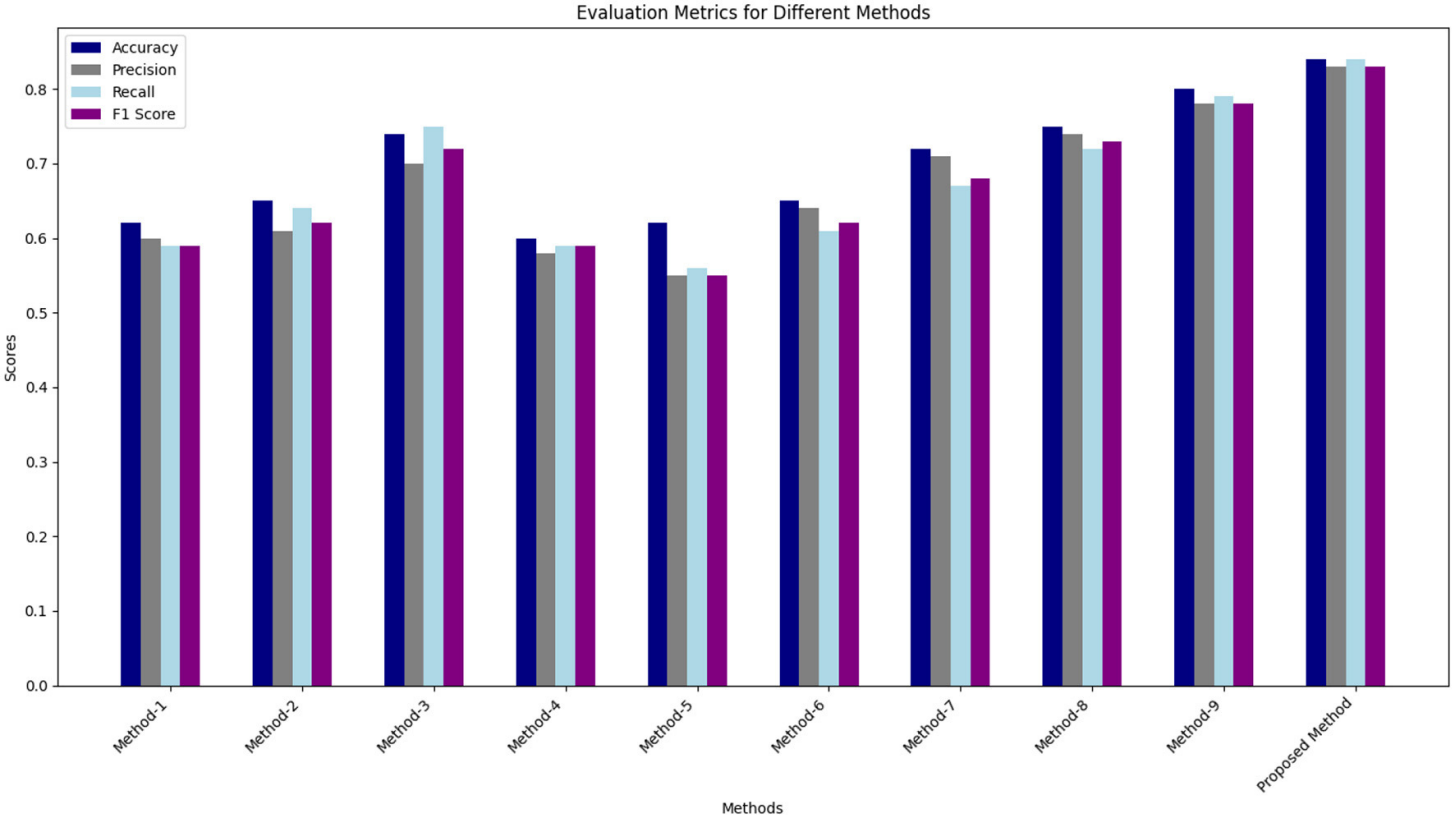
Comparison of results obtained from proposed method with existing methods.

**Figure 8:**
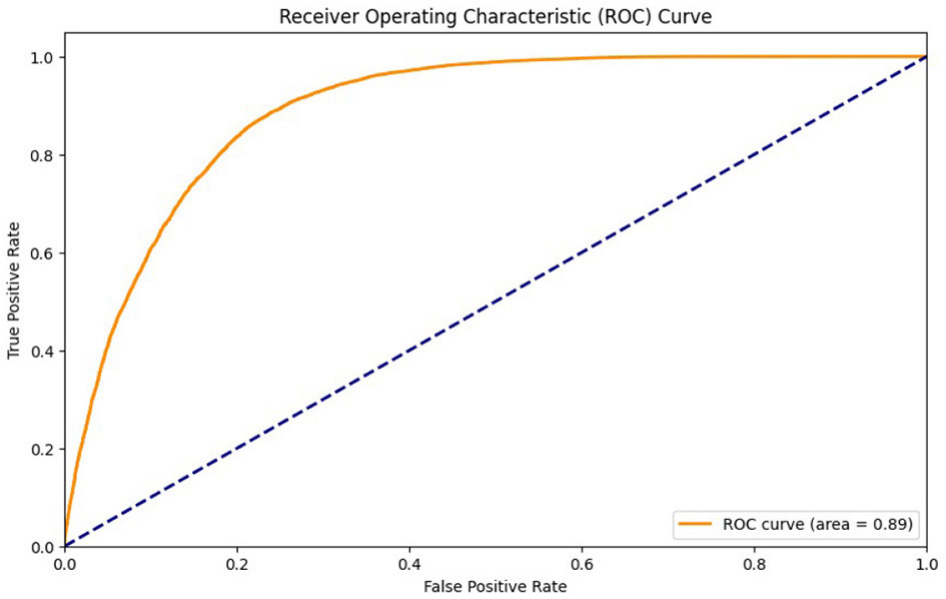
ROC curve of proposed method.

**Figure 9:**
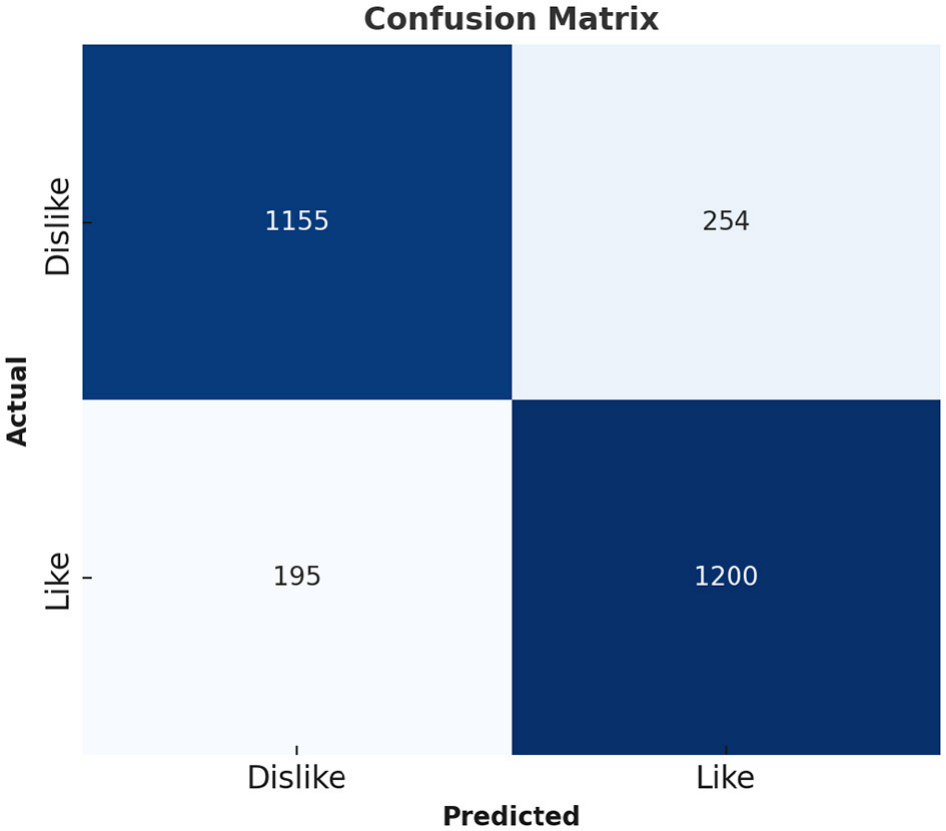
Confusion matrix of proposed method.

Table 6 represents the quantitative comparison of the employed methods, namely accuracy, precision rate, recall, and F1 score. Figures 7–9 show the evaluation score of the method, area under ROC curve, and Confusion Matrix of the proposed method. The ROC curve is a graphical approach that indicates a model's performance at different classification hurdles. It maps True Positive Rate or Sensitivity on the y-axis, against False Positive Rate or Fall out on the x-axis. An ideal ROC curve looks like a graph that plots the data close to the upper left-hand corner of the axes, which means that the performance of the model was satisfactory and it could distinguish between the classes accurately. The AUC gives overall performance of the ROC curve, from this the probability that the model ranks positive instance higher to a randomly chosen negative instance can be determined. Higher AUC shows that the tester has better ability in classifying. AUC-ROC of 0.89 has been achieved as shown in the Figure 8, whereas, confusion matrix is presented in Figure 9 which further proves that it is highly effective when it comes to discriminating between the positive and the negative classes. This score can be classified within the “good” region; hence it can be deduced that the method purposed is good in segregating the two classes of interest.

Figure 7 compares the results obtained from proposed method with the existing state of the art methods. Table 6 describes the evaluation criteria to different methods. As can be seen, the proposed method that uses the given preprocessing for EEG and ET data and incorporates the features of ML as well as DL within stacking ensemble provides the highest results on all of the listed measures.

At the highest accuracy, Method 1 employing raw EEG data with ML features and SVM yielded an accuracy 0.62. At the same time, the proposed method is much more effective with accuracy 0.84. It can be seen that this improvement is universality for precision, recall, and F1 score, more manifesting the advantages of data preprocessing and more successful attempt of the stacking ensemble method combining the ML + DL features. If we compare the methods in which preprocessing was used (e.g., Method 1) with those for which preprocessing was not used (e.g., Method 2), one can see that, in many cases, preprocessing has a positive effect on the performance. For instance, in method number 2, the EEG data is preprocessed and the DL features generates higher percentages of accuracy and recall than in method 1.

Methods which simultaneously utilize both EEG and ET data are superior to the methods based on only one type of data. For example, the feature that incorporates preprocessed EEG and ET data with conventional ML features and DL results in Method 9 has an accuracy of 0.80. This shows when there is an integration of the EEG and ET data it is able to provide better results for the model. The proposed method incorporates stacking ensemble, which also improves the performance of classifiers due to features adopted by this method. This leads to the highest values on all accounts, hence promoting a resilient and efficient model.

**Figure 10:**
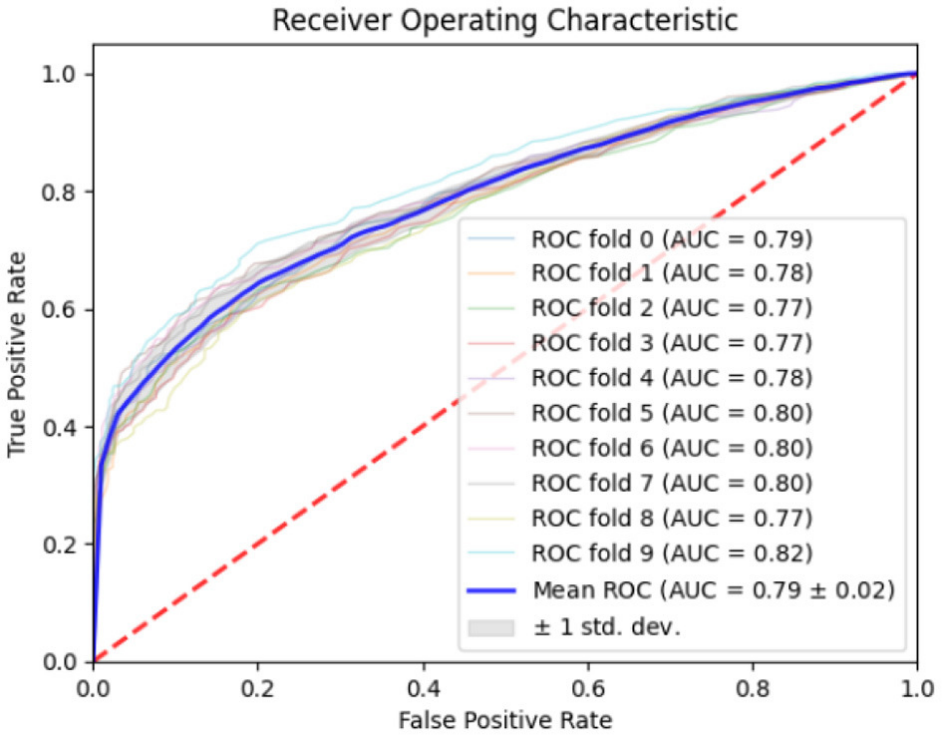
ROC curve of proposed method (EEG only).

Classification results for EEG data when analyzed with the help of ML and DL incorporated with SVM, RF, and DT were seen to be quite satisfactory. By using the approach, the objective was attained with a 0.74 accuracy, and the precision, recall, and the F1 score equal to 0.70%, 0.75%, and 0.72% respectively. Furthermore, the have relatively high AUC, mean of 0.79 as shown in Figure 10 therefore support the reliability and discriminant capacity of the developed model, in the classification of consumers' preferences from EEG signals. Altogether, these metrics can be discussed as demonstrating the efficiency of the proposed approach of applying the traditional ML algorithms alongside with the DL features.

**Figure 11:**
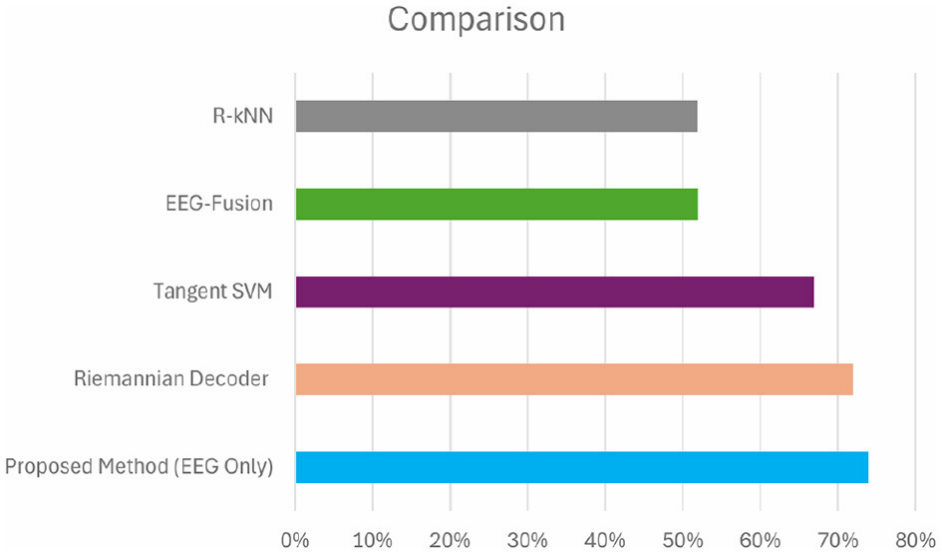
Comparison of accuracy with other methods.

On the other hand, the study that the referenced paper dealt with proposed a new deep learning decoder based on Riemannian Geometry and SPDNet structure (Georgiadis et al., 2023a) for analyzing the signals of the NeuMa dataset of EEG. From the research, the investigators obtained a mean accuracy of 72%. Table 7 shows a comparison between our proposed method(EEG Only) with a state-of-art method of Georgiadis et al. (2023a). The comparison of accuracies is shown in Figure 11. Although this research infuses ML and DL with regular classifiers, the paper's presentation of domain's Riemannian Geometry and SPDNet demonstrates higher accuracy than conventional EEG- based approaches like Tangent Space SVM (Kalaganis et al., 2019), EEG-Fusion (Hakim et al., 2021) and R-kNN (Congedo et al., 2017). The statistical significance thus obtained particularly with reference to the results achieved by Tangent Space SVM which was 67.72%, EEG-Fusion 52.75% and R-kNN was 51.96%.

**Table 7:** Comparison with Georgiadis et al. (2023a)

**Aspect**	**Georgiadis et al. (2023a)**	**Proposed method (only EEG)**
Dataset	NeuMa	NeuMa
Accuracy	0.72	0.74
Precision	Not mentioned	0.70
Recall	Not mentioned	0.75
F1-score	Not mentioned	0.72
AUC score	Not mentioned	0.79

There are some limitations to the study that need to be noted. First off, although 42 participants is a small sample size, it might not be enough to extrapolate the results to a broader population. Furthermore, the findings are predicated on a particular dataset, which can restrict their generalizability to other product categories, markets, and cultural settings. The accuracy of the data acquired may be affected by the sampling rate and precision constraints of the EEG and eye-tracking sensors, despite their effectiveness. Furthermore, even though the used feature extraction strategies which combined manually created and automatically generated features proved successful, more research into different approaches or sophisticated deep learning architectures may enhance model performance. Finally, the integration of EEG and ET data adds complexity to the analysis, and potential synchronization challenges may have influenced the overall accuracy of the model.

## 6 Conclusions

Prediction of consumer preferences that we suggest is based on the machine learning and deep neural network methodology characterized by a high degree of accuracy and precision. These results could have been achieved because of correct preprocessing of images, use of the right features, and the high accuracy classifier. In preprocessing, we have increased the signal-to-noise ratio of EEG signals and ET data by removing noise and balanced the number of samples for classes, specifically the Buy class, by creating more through SMOTE. From the EEG and ET dataset, we created manual features by using the same method as before. Similarly, we used CNN-LSTM for the feature extraction of the selected EEG signals and LeNet-5 for the ET data. In classification, a most dependable stacking classifier was used for classification with a high level of accuracy.

The proposed method demonstrates stable results in the context of consumer preference prediction, though there are opportunities for future studies. For the current extraction feature, we could definitely do better in terms of advanced methodologies such as deep learning architectures or location of brain sources. Classification methods could be enhanced by considering other subject-dependent models or by developing the concept of a prediction. Generalizability is critical, which makes cross-validation mandatory across larger and more diverse data sets. Furthermore, it is necessary to discuss the similarities and differences of the proposed approach with other neuromarketing methods, as well as consider issues of the user's consent and data privacy. In addition, extending this method for uses outside of e-commerce, such as physical store promotion or measuring ad campaign effectiveness, provides more arenas for possible research and practical implementation.

## Data Availability

The original contributions presented in the study are included in the article/supplementary material, further inquiries can be directed to the corresponding author.
